# One crop breeding cycle from starvation? How engineering crop photosynthesis for rising CO_2_ and temperature could be one important route to alleviation

**DOI:** 10.1098/rspb.2015.2578

**Published:** 2016-03-16

**Authors:** Johannes Kromdijk, Stephen P. Long

**Affiliations:** Carl Woese Institute for Genomic Biology, University of Illinois, 1206 Gregory Drive, Urbana, IL 61801, USA

**Keywords:** photosynthesis, mathematical modelling, global change, food supply, genetically modified organisms (GMOs), agriculture

## Abstract

Global climate change is likely to severely impact human food production. This comes at a time when predicted demand for primary foodstuffs by a growing human population and changing global diets is already outpacing a stagnating annual rate of increase in crop productivity. Additionally, the time required by crop breeding and bioengineering to release improved varieties to farmers is substantial, meaning that any crop improvements needed to mitigate food shortages in the 2040s would need to start now. In this perspective, the rationale for improvements in photosynthetic efficiency as a breeding objective for higher yields is outlined. Subsequently, using simple simulation models it is shown how predicted changes in temperature and atmospheric [CO_2_] affect leaf photosynthetic rates. The chloroplast accounts for the majority of leaf nitrogen in crops. Within the chloroplast about 25% of nitrogen is invested in the carboxylase, Rubisco, which catalyses the first step of CO_2_ assimilation. Most of the remaining nitrogen is invested in the apparatus to drive carbohydrate synthesis and regenerate ribulose-1:5-bisphosphate (RuBP), the CO_2_-acceptor molecule at Rubisco. At preindustrial [CO_2_], investment in these two aspects may have been balanced resulting in co-limitation. At today's [CO_2_], there appears to be over-investment in Rubisco, and despite the counter-active effects of rising temperature and [CO_2_], this imbalance is predicted to worsen with global climate change. By breeding or engineering restored optimality under future conditions increased productivity could be achieved in both tropical and temperate environments without additional nitrogen fertilizer. Given the magnitude of the potential shortfall, better storage conditions, improved crop management and better crop varieties will all be needed. With the short time-scale at which food demand is expected to outpace supplies, all available technologies to improve crop varieties, from classical crop breeding to crop genetic engineering should be employed. This will require vastly increased public and private investment to support translation of first discovery in laboratories to replicated field trials, and an urgent re-evaluation of regulation of crop genetic engineering.

## 1. An emerging global food shortage

Human population growth is putting severe pressure on food production to keep up with increasing demand. Two-thirds of calories are derived indirectly or directly from just four crops: rice, wheat, maize and soya bean. In terms of global production of primary foodstuffs, these are the world's top four crops with 741, 716, 1018 and 276 million metric tons, respectively, produced in 2013 [1]. With rising population and changing diet, it is estimated that the world will require 87% more of these primary foodstuffs by 2050 [2]. Production of these crops is strongly dependent on growing season weather and atmospheric conditions, which are predicted to change substantially by mid-century. Although atmospheric [CO_2_] and temperature are consistently predicted to increase, the magnitude of predicted changes in temperature and precipitation vary between the major global circulation models [3,4], with great variation in predictions for the major crop production areas of the globe. In predicting future crop yields, this uncertainty is compounded by great variation between projections from different crop production models even when using just one climate change scenario [5]. Much effort is being placed appropriately into improving both climate and crop production models, to in turn improve certainty about the future state of production of primary foodstuffs. But, at the same time we cannot afford to wait for improved predictions, before preparing for possible futures. Mitigation strategies are available. For example, encourage global diets to become significantly more vegetarian, facilitate improvement of crop yields of farmers in poorer nations towards those of farmers in the developed nations, improve food storage conditions, and reduce waste in the food supply chain. All of these could lower the risk of projected future food shortages. However, given uncertainty in achieving these strategies it behoves national governments to insure against the serious risk that demand for food will continue to outpace production and that this will be exacerbated by global climate change. Emerging food shortages of the 1960s were alleviated primarily by the successes of the Green Revolution, which resulted largely from genetic improvement of the major food crops, and agronomy to support the new crop cultivars. If genetic improvement is again to have a major role in averting today's emerging food shortages, time constraints need to be appreciated. Any genetic innovation, whether by conventional breeding or through genetic engineering, achieved today is unlikely to be in farmers' fields at scale for 30 years. Considering the projected food demands for 2050 and possible climate change impacts, we may therefore state ‘one crop breeding cycle from possible starvation’. That is all the time we have to provide better adapted, more productive crops by 2050. Finally, increasing crop productivity per unit land area is in itself important in avoiding additional greenhouse gas emissions. If prices of primary foodstuffs continue to rise in real terms, as they have in the last decade owing to demand outpacing production, this risks incentivizing expansion of food crop production onto environmentally sensitive land [6], which will accelerate erosion and deforestation, and in turn emissions through land use change.

## 2. Increasing photosynthesis to improve crop productivity

**Figure 1.**
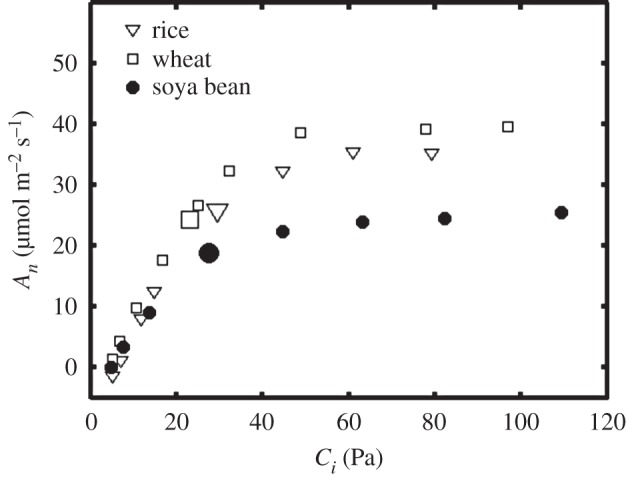
Net leaf assimilation rate (*A_n_*) in saturating light as a function of CO_2_ partial pressure in the intercellular air space (*C_i_*). Example curves for rice (triangles), wheat (squares) and soya bean (circles) were redrawn, respectively, from the following sources [13–15]. Enlarged symbols show the net leaf assimilation rates when the external CO_2_ partial pressure equals that of the current atmospheric level of 39–40 Pa.

Assuming that historic rates of improvement in the yield per hectare of land are maintained through this century; then by 2050, there will be a shortfall of 28% in the amount of rice produced globally, 30% in wheat and 23% in soya bean [2]. But current evidence suggests that even this staggering scale of shortfall could be over-optimistic on what can really be produced. First, over the last decade, it has become apparent that historic rates of increase are not being maintained, with yield increases over the past decade in these crops being a quarter of that of the Green Revolution years for rice and near zero for wheat [7–9]. Climate change [10] and rising tropospheric ozone [11] may play some part in this, but the greater effect is owing to the fact that genetic changes achieved during the Green Revolution, notably improvement in the harvest index, the proportion of plant biomass that is partitioned into the harvested product (e.g. grain of rice), is close to its biological limit. Interestingly, the conversion efficiency of absorbed sunlight into plant biomass, i.e. crop photosynthesis, has not been improved substantially over the same period. It is perhaps surprising that traditional plant breeding has failed to improve this conversion efficiency. Three main reasons can be put forward to explain this [12]. First, as instrumentation to measure leaf photosynthesis became available in the 1960s and 1970s, the measured rates showed little to no correlation with crop yield. Second, the yield of major food crops appeared to be limited not by the production of assimilates, but by their genetic potential to develop sufficient numbers of sink organs, such as the seeds of soya bean, grains of rice or tubers of potato. Third, it was reasoned that if photosynthesis is a key determinant of yield, then selection by breeders would have resulted inadvertently in crops with increased photosynthesis. So what has changed this paradigm? Rather cynically, the substantial body of research into the effects of global climate change on plant growth has provided the most compelling evidence to rethink the role of photosynthesis in final crop yield. Global climate change is dominated by rising [CO_2_]. Recognition of this key environmental change spurred a wide range of experiments that investigated the direct effects of elevated [CO_2_] on crops. In C_3_ crops such as rice, wheat or soya bean, the direct effect of elevated [CO_2_] is to accelerate photosynthetic rate and to suppress photorespiration, causing an increase in net photosynthesis (figure 1).

Strong support for the hypothesis that these increases in photosynthesis translate in yield comes from Free-Air [CO_2_] Enrichment (FACE) studies. Here, a crop is grown from sowing to harvest under fully open-air elevation of [CO_2_] to forecast future conditions. In FACE experiments, the increases in photosynthesis translated into significant yield increases for conventional wheat, rice and soya bean cultivars of approximately 15%. This however, was only half the theoretical level [16]. In theory, elevation of [CO_2_] from current to 550 ppm could potentially increase net carbon gain and yield by *ca* 30%, and FACE experiments with a new hybrid rice cultivar [17] arrive at exactly this number. Additionally, despite their record yields, new super hybrid rice lines show poor filling of caryopses in their inferior spikelets [18]. Both results suggest strong source limitation in these most recent cultivars. So, while the seed as sink for photosynthate was once considered the major barrier to increasing crop yields, this appears no longer the case, suggesting that further genetic improvement in yield potential will require increased photosynthetic capacity.

Source is determined by the integrated photosynthesis of the whole crop, less respiratory losses. What prospects are there for adapting photosynthesis, and hence source, in crops to global change? Here we focus on two aspects of global change, rising [CO_2_] and rising temperature, arguably the most certain of changes as we move into a greenhouse future. The Intergovernmental Panel on Climate Change (IPCC) Fifth Assessment Report predicts that by 2050, with ‘business-as-usual’, the [CO_2_] will have reached 55 Pa and average surface air temperature will have risen by 1.65°C [4]. In the following paragraphs we explore, from the underlying biochemical and biophysical principles, how leaf and crop photosynthesis respond to rising [CO_2_], how this would be modified by rising temperature, and how this knowledge may be exploited to develop more productive crop cultivars.

## 3. CO_2_ sensing

Crops may only perceive a change in [CO_2_] directly through tissues that are exposed to the open air. With the exception of some reproductive organs, only the photosynthetic tissues have any direct contact with the atmosphere. The cuticle of crop leaves and other photosynthetic organs determines that only the inner surfaces of the guard cells of the stomata and the mesophyll can directly sense a change in atmospheric [CO_2_]. While many steps in metabolism use CO_2_ or are affected by its concentration, the only convincing evidence for a response in the concentration range of relevance to contemporary atmospheric change (24–75 Pa) are at the level of the primary carboxylase of C_3_ photosynthesis, Rubisco (ribulose-1:5 bisphosphate carboxylase/oxygenase) and a metabolic step affecting stomatal aperture [19,20].

**Figure 2.**
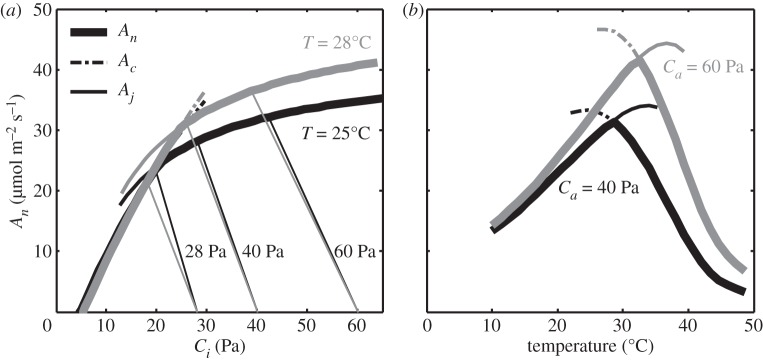
(*a*) CO_2_ response of net assimilation rate (*A*_*n*_) for leaf temperature (T) of 25°C (black) or 28°C (grey). Supply functions are shown to depict stomatal limitation for ambient CO_2_ partial pressure (*C*_*a*_) at 28 Pa (preindustrial), 40 Pa (current) and 60 Pa (future predictions). (*b*) Temperature response of net assimilation rate (*A*_*n*_) at ambient CO_2_ partial pressure of 40 Pa (black) or 60 Pa (grey). *V*_cmax_ = 130 µmol m^−2^ s^−1^, *J*_max_ = 205 µmol m^−2^ s^−1^ [25].

Stomatal apertures and therefore leaf conductance decrease in response to rising [CO_2_] so it might be expected to decrease the magnitude of increase in the CO_2_ partial pressure in the intercellular air space (*C_i_*). However, an analysis of C_3_ plants growing in current and future [CO_2_] under fully open-air FACE conditions showed no change in the ratio of leaf intercellular air space to atmospheric [CO_2_] (*C_i_*/*C_a_*), which remained remarkably constant at approximately 0.71 [21]. Only Rubisco has both the potential to respond markedly to increasing [CO_2_] and is a key metabolic step with sufficient regulatory control that a change in reaction rate would alter the flux through a major metabolic pathway. The increase in photosynthesis owing to elevation of [CO_2_] results from two properties of Rubisco in C_3_ plants: (i) the K_m_ of the enzyme for CO_2_ is close to the current atmospheric concentration, so the velocity of carboxylation is increased by elevated [CO_2_]; and (ii) the oxygenation reaction, which produces glycolate leading to photorespiration, is competitively inhibited by CO_2_. At low [CO_2_], photosynthesis is limited by the activity of Rubisco, and so photosynthesis benefits from both properties. Under conditions where photosynthesis is not limited by the activity of Rubisco but by the supply of the substrate for carboxylation and oxygenation, ribulose bisphosphate (RuBP), the latter effect will still serve to increase photosynthesis. This is apparent in figure 1, explaining the rapid increase in *A*_*n*_ with the initial increase in *C*_*i*_, and a slower increase in *A*_*n*_ with further increase in *C*_*i*_ above the current atmospheric [CO_2_] of *ca* 40 Pa. This latter effect is of significance since it serves to increase the efficiency of net CO_2_ uptake by decreasing photorespiratory CO_2_ loss, reducing ATP and NADPH demand for photorespiratory metabolism and so making more of these products of the light reactions of photosynthesis available for CO_2_ assimilation. As a result, even under limiting light, as for example in the lower canopy of crops, elevated [CO_2_] will boost crop carbon uptake. What is the relative contribution of these two properties? Assuming an average specificity and K_m_ for CO_2_ and O_2_ for Rubisco from terrestrial plants, the increases in net CO_2_ uptake that would result from increase in atmospheric [CO_2_] may be calculated by coupling two simple models for stomatal conductance [22] and photosynthesis [23,24]. At low *C_i_* (rule of thumb less than 30 Pa) photosynthetic CO_2_ fixation is modelled to be limited by *V*_cmax_, the maximal carboxylation rate of Rubisco. By contrast, at high values of *C_i_* (more than 40 Pa) regeneration of the primary substrate RuBP controls CO_2_ uptake, which in the model is derived from the maximal rate of linear electron transport (*J*_max_). As a result, the dose–response curve over the full range of CO_2_ concentrations can be simulated by two intersecting Michaelis–Menten curves (*A_c_* and *A_j_*, see figure 2*a*), which both have specific temperature response functions (figure 2*b*). It is worth mentioning that in some cases, a third equation has been added to describe the rate-limiting effect of triose-P usage [26] at very high values of *C_i_*. Using this model, for a leaf temperature of 25°C, the increase in atmospheric [CO_2_] from today's 40 to 55 Pa by the mid-century would increase Rubisco-limited and RuBP-limited leaf photosynthesis by 33% and 11%, respectively (for a full description of the equations, see the electronic supplementary material).

## 4. Optimizing photosynthetic leaf nitrogen allocation

Keeping with the nomenclature of this widely used photosynthesis model, both *V*_cmax_ and *J*_max_ represent a leaf nitrogen investment and the resulting trade-off makes photosynthetic nitrogen use efficiency optimal at the value of *C_i_*, where *A_c_* and *A_j_* intersect, also termed the inflection point. Owing to the steeper temperature response of RuBP-oxygenation compared to RuBP-carboxylation, the inflection point increases with temperature (figure 2*a*) and competitive inhibition of oxygenation by high CO_2_ leads to an increase in optimal temperature for leaf photosynthesis (figure 2*b*). Figure 2*a* also shows CO_2_ supply functions that are determined by stomatal conductance, for [CO_2_] representative of preindustrial (28 Pa), current (40 Pa) and that predicted for 2050 (60 Pa). At 28 Pa, leaf photosynthesis is primarily limited by RuBP-carboxylation, but at 40 Pa and especially at 60 Pa leaf photosynthesis becomes progressively more limited by RuBP-regeneration. Since the resulting overcapacity in Rubisco reduces photosynthetic nitrogen use efficiency, re-allocation of leaf nitrogen from *V*_cmax_ to *J*_max_ would improve leaf photosynthesis under increasing [CO_2_] without using more nitrogen. However, current C_3_ crops often appear unable to gain the full advantage of this optimization. This may have evolutionary origins. The average [CO_2_] over the past 25 Myr was relatively stable at 28 Pa [27]. Considering that photosynthesis, especially at this low [CO_2_], must have represented a strong selective pressure for survival and reproduction in many environments, evolution has had millions of generations to adjust to pre-industrial [CO_2_], whereas the recent anthropogenically driven increase in [CO_2_] has lasted little over 100 generations for most annual higher plants, far fewer for most perennials. Global climate change is simply happening too fast for evolutionary adjustment to keep up. It is therefore no surprise that computational analysis suggests investment in photosynthetic enzymes and machinery to be sub-optimal for current [CO_2_] and even more so for future [CO_2_] [28].

**Figure 3.**
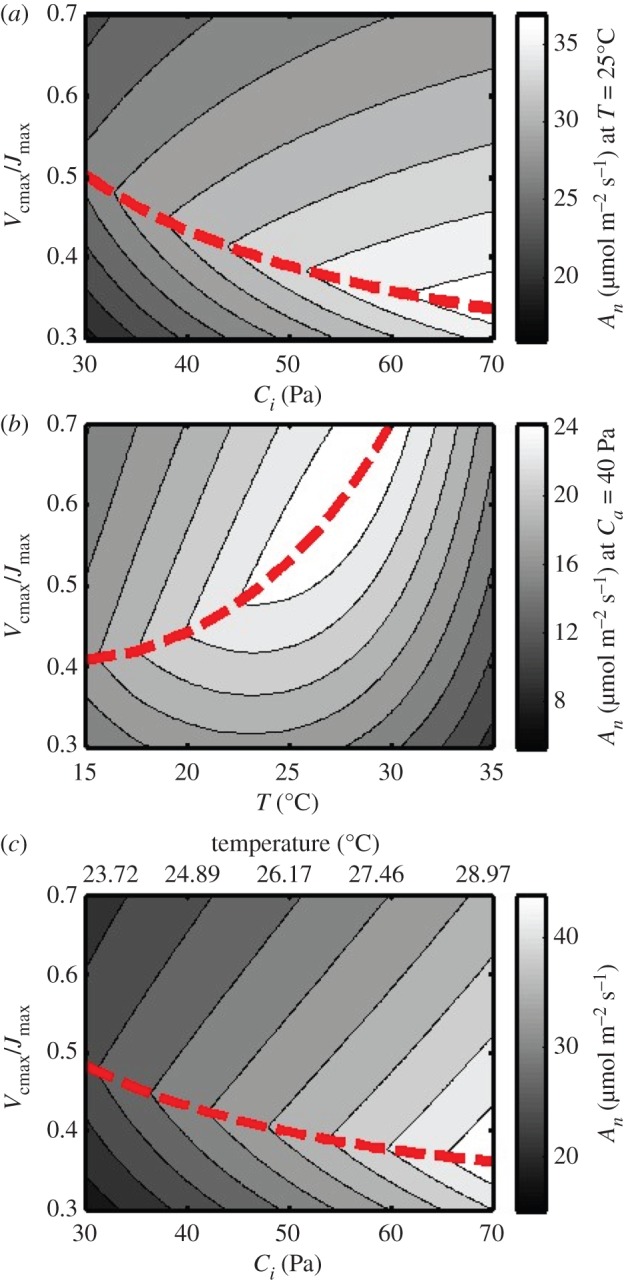
(*a*) Net CO_2_ assimilation rate (*A_n_*) as a function of: (*a*) intercellular CO_2_ partial pressure *C_i_* (Pa) and varying *V*_cmax_/*J*_max_ at constant leaf temperature of 25°C. (*b*) Net CO_2_ assimilation rate as a function of leaf temperature *T*(°C) and varying *V*_cmax_/*J*_max_ at constant ambient CO_2_ partial pressure of 40 Pa. (*c*) Net CO_2_ assimilation rate as a function of *V*_cmax_/*J*_max_ and covarying leaf temperature *T* (°C) and intercellular CO_2_ partial pressure *C_i_* (Pa). Temperature was covaried with ambient [CO_2_] according to a linear regression (*r*^2^ = 0.992) between predicted future CO_2_ increases and temperature anomalies derived from IPCC predictions (2013). In all simulations leaf nitrogen content was kept constant at 2 g N m^−2^. The red broken line indicates optimal *V*_cmax_/*J*_max_ ratio for each *C_i_* or *T*, i.e. where *A_n_* reaches the highest value and photosynthetic nitrogen use efficiency peaks.

When we used the model to calculate optimal *V*_cmax_/*J*_max_ (assuming constant leaf temperature and photosynthetic nitrogen content), it was observed to decline with increasing CO_2_ (figure 3*a*). However, the predicted increase in atmospheric CO_2_ is associated with concomitant increases in air- and associated leaf temperature (T), which have the opposing effect on optimal *V*_cmax_/*J*_max_ (figure 3*b*). When both [CO_2_] and *T* are covaried together according to a linear regression (*r*^2^ = 0.992) based on the most recent IPCC predictions (see the electronic supplementary material), it is clear that the optimal *V*_cmax_/*J*_max_ will decline owing to a stronger influence of [CO_2_], although the decline is somewhat less pronounced at very high values of *C_i_* (figure 3*c*).

## 5. Will leaf-level advantages in optimizing *V*_cmax_/*J*_max_ persist at canopy scale?

**Figure 4.**
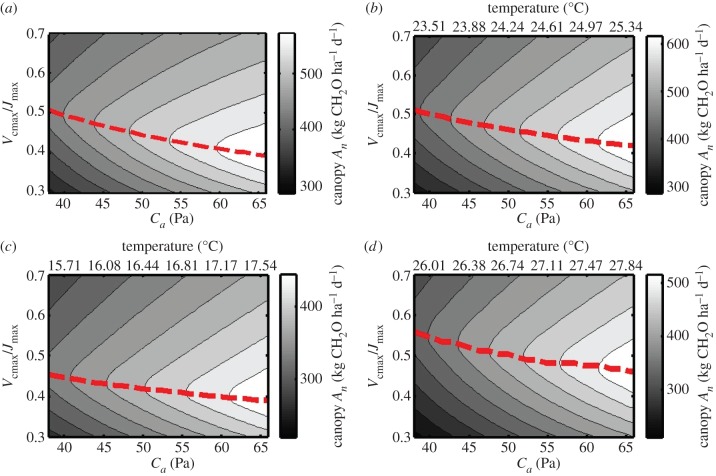
Canopy diurnal photosynthetic carbohydrate production as a function of atmospheric CO_2_ partial pressure (*C_a_*) and varying *V*_cmax_/*J*_max_ at a constant temperature of 25°C (*a*) or covariation of temperature as a function of *C_a_* (*b*–*d*). Computations were performed for three geographical locations: (*a*,*b*) Midwest USA (42.5° N, 92.5° W), (*c*) UK (52° N, 0° E) and (*d*) Uganda (0.3° N, 32.6° E) for simulated light and temperature conditions on 31 July. Temperature was covaried with *C_a_* according to a linear regression (*r*^2^ = 0.992) between predicted future [CO_2_] increases and temperature anomalies derived from IPCC predictions (2013). For further details, see the electronic supplementary material.

The simulations in figure 3*a*–*c* show that the covariation between rising [CO_2_] and temperature increase will determine if and in which direction *V*_cmax_/*J*_max_ should be changed to improve leaf photosynthesis. The actual increase in *C_i_* will be dampened relative to atmospheric [CO_2_] owing to the regulatory role of stomatal conductance, which declines with increasing [CO_2_]. Additionally, the simulations in figure 3 assume saturating light and are therefore only representative of a subset of leaves at the top of the canopy for a limited part of the diurnal period. Since more than 50% of diurnal canopy photosynthesis potentially occurs under light-limiting conditions [29], will the optimal *V*_cmax_/*J*_max_ still decline when scaling up to canopy level? These questions were addressed with a simple canopy photosynthesis model in figure 4 (for equations see the electronic supplementary material). Figure 4*a* shows the change in optimal *V*_cmax_/*J*_max_ for canopy photosynthesis as a function of changing atmospheric [CO_2_] (*C_a_*). The simulated values of canopy photosynthesis were computed for a fully developed canopy and integrated over one diurnal cycle in the middle of the growing season of the United States Midwest (for full details, see the electronic supplementary material). Figure 4*a* clearly shows that scaling up to canopy level does not affect the trend of a declining optimal *V*_cmax_/*J*_max_ with increasing [CO_2_], even though a substantial part of canopy photosynthesis takes place under light-limited conditions. But what about the effect of increasing temperature? This may depend on location. In cool climates, a temperature increase is expected to boost photosynthesis, while it could become supra-optimal and stressful in a hot climate. Figure 4*b*–*d* shows the simulation for three different geographical locations: figure 4*b* (42.5° N, 92.5° W, Midwest USA), figure 4*c* (52° N, 0° W, UK) and figure 4*d* (0.3° N, 32.6° E, Uganda) where temperature is simulated to covary according to the global predictions by the IPCC. In other words, the *x*-axes in figure 4*b*–*d* represent the predicted future increase in both atmospheric [CO_2_] and temperature. Figure 4*b*–*d* thus account for the opposing effect of rising temperature on optimal *V*_cmax_/*J*_max_, as well as associated dampening of the increase in *C_i_* through adverse effects of temperature-induced increases in vapour pressure deficit on stomatal conductance, assuming that absolute vapour pressure remains constant. However, optimal *V*_cmax_/*J*_max_ for canopy carbon gain still strongly declines under all simulated future climate conditions, in both temperate and tropical conditions. In summary, these results suggest that under all conditions a shift in allocation of resources from Rubisco to the apparatus regenerating RuBP would increase photosynthesis without requiring any additional resource.

## 6. How can increases in *V*_cmax_*/J*_max_ be achieved?

According to the simulations in figure 4*b*–*d*, future crops could benefit from higher *J*_max_ at the expense of *V*_cmax_. So what tools are available to effect this change? Freeing up leaf nitrogen from Rubisco, represented by a decline in *V*_cmax_, may be achieved by tapping into existing acclimation responses. Results from a cross-comparison of FACE studies show that acclimation to elevated [CO_2_] often results in a stronger decline of *V*_cmax_ compared to *J*_max_, along with decreases in total leaf nitrogen content and accumulation of leaf non-structural carbohydrates [21], leading to a small (5%) but significant reduction in *V*_cmax_/*J*_max_. However, this adjustment does not appear to go far enough. Photosynthetic parameters in soya bean, grown at elevated [CO_2_] and temperature in a cool (2009) and hot (2011) summer, were measured in an open-air treatment facility [25]. Comparing observed to predicted optimal *V*_cmax_/*J*_max_ (table 1) showed a consistent over-investment in Rubisco, represented by *V*_cmax_. This led to an average reduction of predicted canopy productivity of 12.7% across treatments and years to that which could be achieved with an optimal distribution of resources between these two potential limitations.

**Table 1.** Observed and optimal *V*_cmax_/*J*_max_ ratios for soya bean grown at elevated [CO_2_], air temperature or both and associated predicted reductions in canopy productivity. (Observed parameter values were obtained from [25]. Results are shown for two contrasting field seasons in Illinois: 2009 (cool) and 2011 (hot).)

year	[CO_2_] (Pa)	monthly average *T* (°C)	Δ*T* (°C)	observed *V*_cmax_/*J*_max_	optimal *V*_cmax_/*J*_max_	predicted canopy productivity with observed *V*_cmax_/*J*_max_ ratio (kg CH_2_O m^−2^ d^−1^)	predicted canopy productivity with optimal *V*_cmax_/*J*_max_ ratio (kg CH_2_O m^−2^ d^−1^)	relative productivity difference optimal versus observed *V*_cmax_/*J*_max_ ratio (%)
2009	38.5	21.3	0	0.63	0.47	383	421	10
2009	38.5	21.3	3.5	0.72	0.54	399	446	12
2009	58.5	21.3	0	0.63	0.40	453	525	16
2009	58.5	21.3	3.5	0.66	0.44	513	590	15
2011	38.5	27.2	0	0.79	0.60	397	448	13
2011	38.5	27.2	3.5	0.81	0.72	395	411	4
2011	58.5	27.2	0	0.71	0.48	541	623	15
2011	58.5	27.2	3.5	0.79	0.57	559	640	14

Whereas the acclimation response of *V*_cmax_*/J*_max_ arises mainly from the decline of *V*_cmax_, using this release of leaf nitrogen to increase investment in *J*_max_ may prove more difficult to achieve. Considerable genetic variation in *V*_cmax_ and *J*_max_ is present in the existing germplasm for wheat, rice and soya bean [14,30,31], but owing to significant correlation between both parameters, *V*_cmax_/*J*_max_ tends to be more conserved. Interestingly, reduction of Rubisco content in rice via antisense expression of the small subunit sequence was accompanied with non-specific increases in thylakoid components and higher rates of CO_2_-saturated photosynthesis [32,33]. However, similar Rubisco reductions in tobacco did not affect expression of other photosynthetic components [34] and targeted changes through transgenic manipulation may therefore be necessary to achieve increases in *J*_max_. The cytochrome b6f complex was shown to strongly control the rate of RuBP-regeneration in plants with reduced Rieske FeS protein content [35,36], but overexpression efforts have thus far been unsuccessful. Modelling analysis has identified a number of other proteins, which may also share control over *J*_max_ and carbon assimilation at high CO_2_ and these are currently subject to extensive evaluation [28,37]. One particularly promising line of work arose from the finding that the Calvin–Benson–Bassham cycle enzyme SBPase also exerts considerable control over RuBP-regeneration capacity [38,39]. Transgenic overexpression of SBPase was shown to increase *J*_max_, photosynthetic capacity and growth of tobacco plants [39,40]. These findings were confirmed in open-air field experiments which showed the stimulation of biomass yield of the SBPase overexpression lines was greater under elevated [CO_2_] [25]. Thus, there is proof-of-concept that manipulation of a single enzyme can actually enhance carbon assimilation and yield under future climate conditions, in line with model predictions.

## 7. Concluding remarks and future directions

In the preceding paragraphs, we have provided an example strategy to better adapt crops to the predicted global change in [CO_2_] and temperature. A strategy which shows that by targeting the primary process that drives crop production, i.e. photosynthesis, increased calories per hectare could be achieved under global atmospheric and climatic change, and achieved sustainably. In this particular example, the proposed strategy would have most effect when crop yield approaches potential yield, i.e. in the absence of other limitations to yield, such as pests, diseases or resource limitation. Under these conditions, crop yield would be mostly limited by photosynthesis. When conditions are less favourable for crop growth, improvement of photosynthesis should still increase crop yield since efficiency is improved, i.e. the crop becomes more water and nitrogen use efficient [41]. For conditions of water and nutrient limitation, it would be advisable to re-assess the optimal *V*_cmax_/*J*_max_ calculations, including any additional limitations that are deemed relevant, to estimate the potential yield increase through optimization of *V*_cmax_/*J*_max_. The model calculations of [CO_2_] and temperature interactions should also be seen in this context, since we only considered [CO_2_] and temperature effects on photosynthesis, excluding any other processes that may also be affected. For example, when rising temperature exceeds thresholds for the normal development of flowers, for pollination, for seed filling or for other processes essential to normal crop development, far more deleterious effects on yields can be expected that will require other strategies [42,43]. It also remains to be seen to what extent a central process like photosynthesis can be altered, without having to adjust various auxiliary processes to maintain balanced and resilient crop growth [44]. The modelling in the previous paragraphs was kept at its basics to demonstrate the point, but more refined simulation models, integrating photosynthetic responses with crop development from gene expression to growth over the growing season, will be able to more accurately predict the benefits of any optimization strategy for different crops and environments. However, all these refinements and nuances aside, strategies targeted to adapt crops for higher productivity at future climate conditions are described in many papers [45–47], but progress in transferring ideas or proof-of-concepts to real crop improvement has so far been limited. This transition phase lies beyond the scope of most academic research laboratories and represents by far the most expensive, yet critical, phase in the development of new crop varieties. In the case of transgenic crops, the costs are especially inflated owing to seemingly futile discussions about applications of recombinant DNA technology in crop improvement leading to counterproductive regulation [48]. In the face of the extra-ordinary challenges ahead, we simply do not have the luxury to rule out the use of any technology that may hold promise to improve crop performance. What we need now is an increased unhindered effort to investigate, execute and fully test and verify promising crop improvement strategies, from model simulations and controlled laboratory conditions to fully replicated field trials.

## Supplementary Material

Whole crop photosynthesis model
