# Supplementary material for: One crop breeding cycle from starvation? How engineering crop photosynthesis for rising CO2 and temperature could be one important route to alleviation
Source: Proc Biol Sci. 2016 Mar 16;283(1826):20152578. doi: 10.1098/rspb.2015.2578 (PMC4810849; doi:10.1098/rspb.2015.2578)
Supplement: Whole crop photosynthesis model [file rspb20152578supp1.docx]

**Leaf photosynthesis**

Leaf photosynthesis was calculated following Long and Bernacchi (2003) and assumed to be either limited by Rubisco carboxylation (Eq1) or linear electron transport (*J*, Eq2). For simplification, limitation by triose phosphate use was not considered.

1. $W_{C}=\frac{V_{cmax}C_{i}}{(C_{i}+K_{C}(1+\frac{O}{K_{O}})}$
2. $W_{J}=\frac{JC_{i}}{({4C}_{i}+8\Gamma^{*})}$

Where:

1. $J=\frac{Q_{2}+J_{max}-\sqrt{{{(Q}_{2}+J_{max)}}^{2}-4\theta_{PSII}Q_{2}J_{max}}}{2\theta_{PSII}}$
2. $Q_{2}=PFD\alpha_{l}\Phi_{PSII,max}\beta$

For complete description and values of constants and parameters, see Table S1. Based on Eq1-4, net assimilation rate can be computed as:

1. $A_{n}=\min\left( W_{C},W_{J} \right)\left( 1-\frac{\Gamma^{*}}{C_{i}} \right)-R_{d}$

Stomatal conductance was computed as a function of ambient CO_2_ (*C_a_*), relative humidity (*H_r_*) and net assimilation rate (*A_n_*) using the Ball-Berry model (Ball *et al.* 1987):

1. $g_{s\_H2O}=g_{0}+\frac{g_{1}A_{n}H_{r}}{C_{a}}$

The stomatal conductance to CO_2_ (g_s_) was computed by dividing *g_s_H2O_* (from Eq.6) by 1.6 to account for the difference between H_2_O and CO_2_ molecular diffusivities. Then, following Fick’s law, *A_n_* can also be described by Eq.7:

1. $A_{n}=g_{s}(C_{a}-C_{i})$

An iterative loop was used to find the value of intercellular CO_2_ partial pressure (*C_i_*) where equation 5 and 7 yield equal values for *A_n_*.

Temperature corrections were applied according to Eq.8 and Eq.9 (Sharkey *et al.* 2007):

1. $K_{C}=e^{\left( 35.9774-\frac{80.99}{0.008314\left( 273.15+T \right)} \right)}$

$$K_{O}={1000e}^{\left( 12.3772-\frac{23.72}{0.008314\left( 273.15+T \right)} \right)}$$

$$\Gamma^{*}={\frac{O}{21000}e}^{(11.187-\frac{24.46}{0.008314\left( 273.15+T \right)})}$$

1. For rate parameters the parameter value at 25^o^C was multiplied with the following correction factors:

$$corrV_{cmax}=e^{(26.355-\frac{65.33}{0.008314\left( 273.15+T \right)})}$$

$$corrR_{d}=e^{(17.71-\frac{43.9}{0.008314\left( 273.15+T \right)})}$$

$$corrJ=e^{(18.715-\frac{46.39}{0.008314\left( 273.15+T \right)})}$$

**Distribution of leaf nitrogen between Jmax and Vcmax**

*V_cmax_* and *J_max_* were previously described as a function of total leaf nitrogen content by Harley *et al.* (1992):

1. $V_{cmax}=60N_{tot}-9.6$
2. $J_{max}=98.1N_{tot}-4.6$

These correlations (Eq.10 and Eq.11) describe *V_cmax_* and *J_max_* as a function of total leaf N (*N_tot_*). We want to simulate different *V_cmax_*/*J_max_* ratios while keeping total leaf N constant. We therefore redistribute a fraction of leaf photosynthetic N between *V_cmax_* (*f_NV_*) and *J_max_* (*f_NJ_*) which has to be considered mutually exclusive in order to keep total N constant (Eq.12):

1. $f_{NV}=-f_{NJ}$

Incorporation of *f_NV_* and *f_NJ_* in Eq.10 and Eq.11 then gives:

1. $V_{cmax}={60N}_{tot}(1+f_{NV})-9.6$
2. $J_{max}={98.1N}_{tot}(1+f_{NJ})-4.6$

**Canopy photosynthesis**

Canopy photosynthesis was computed following Ort *et al.* (2010). First, the canopy leaf area (*F_tot_*) was divided in sunlit (*F_sun_*) and shaded (*F_shade_*) leaf area (Eq.15-17), as a function of the foliar extinction coefficient (*k*, Eq.18), the solar zenith angle (θ, Eq. 19) and the solar declination (*δ*, Eq. 20).

1. $F_{tot}=F_{sun}+F_{shade}$
2. $F_{sun}=\frac{(1-e^{-kFcos\left( \theta\right)})cos(\theta)}{k}$
3. $F_{shade}=F_{tot}-F_{sun}$
4. $k=\frac{cos(\theta)\sqrt{x^{2}+{tan}^{2}\left( \theta\right)}}{{x+1.744(x+1.882)}^{-0.733}}$
5. $\cos\left( \theta\right)=\sin\left( \Omega\right)\sin\left( \delta\right)+\cos\left( \Omega\right)\cos\left( \delta\right)cos(15\left( t-t_{sn} \right))$

Where Ω, *t* and *t_sn_* represent the latitude, time of day (hr) and time of solar noon (hr), respectively. x represents the ratio of horizontal to vertical projected canopy leaf area. The solar declination is represented by *δ* and computed by Eq. 20:

1. $\delta=-23.5cos(\frac{360\left( D_{j}+10 \right)}{365})$

In which *D_j_* represents the day of year.

Irradiance was subsequently divided in direct and diffuse components according to Eq. 21 and 22:

1. $I_{direct}=I_{S}\alpha^{\frac{P/P_{o}}{cos(\theta)}}$
2. $I_{diff}=0.5I_{S}(1-\alpha^{\frac{P/{P_{o}}}{\cos\left( \theta\right)}})cos(\theta)$

Scattered irradiance was computed according to Eq.23:

1. $I_{scatt}=0.07I_{direct}\left( 1.1-0.1F \right)e^{-cos(\theta)}$

Eq. 24 and 25 were then used to compute the incident PFD on the sunlit (*I_sun_*) and shaded part of the canopy (*I_shade_*):

1. $I_{shade}=I_{diff}e^{(-0.5F^{0.7})}+I_{scatt}$
2. $I_{sun}=\frac{I_{direct}\cos\left( \lambda\right)}{\cos\left( \theta\right)}+I_{shade}$

In which *λ* represents the angle between the leaf surface and the direct component of incident PFD:

1. $\lambda=\cos^{-1}(k)$

Incident PFD for sunlit and shaded leaf area was used to compute net assimilation rate at leaf-level for both components (*A_sun_*, *A_shade_*) according to Eq.1-14. Finally, to compute diurnal canopy photosynthesis, sunlit and shaded contributions were summed according to:

1. $A_{canopy}=\sum_{1}^{24} {(A}_{sun}F_{sun}+A_{shade}F_{shade})$

**Canopy photosynthesis as a function of predicted climate change**

When canopy photosynthesis was computed as a function of two variables (i.e. CO_2_ and *V_cmax_*/*J_max_* or *T* and *V_cmax_*/*J_max_*), responses were computed according to the above-mentioned equations, where leaf temperature was assumed to be equal to air temperature.

When we included temperature as a co-factor in the response of canopy photosynthesis to CO_2_ and *V_cmax_*/*J_max_* we applied linear regression to IPCC (Collins *et al.* 2013) simulations for global temperature anomalies (*T_Δ_*) and atmospheric CO_2_ concentrations (*C_a_*) between 1850-2100, yielding the following correlation (R^2^=0.992):

1. $T_{\Delta}=0.0073Ca-2.6063$

We used a look-up table (HadCRUT, Jones *et al.* 1999) to find the average absolute temperature between 1961-1990 as a function of latitude, longitude and month of year for specified spatial coordinates. Finally, *T_Δ_* from Eq. 28 was then used to compute the absolute temperature as a co-factor with atmospheric CO_2_.

When computing temperature effects on photosynthesis, absolute vapour pressure was kept constant and approximated using an input value of relative humidity *H_r_* at 25 ^o^C, and the approximation of saturated water vapour pressure *e_s_* using the polynomial by Flatau *et al.* (1992):

1. $e_{s}=C_{1}+C_{2}T+C_{3}T^{2}+C_{4}T^{3}+C_{5}T^{4}+C_{6}T^{5}+C_{7}T^{6}$

In which:

C_1_ = 6.11176750, C_2_ = 0.443986062, C_3_ = 0.0143053301, C_4_ = 0.000265027242,

C_5_ = 0.00000302246994, C_6_ = 0.0000000203886313, C_7_ = 0.0000000000638780966

**Table S1 Description of parameters and constants.** Values used are between parentheses, unless specified otherwise in the text.

| **Name (value)** | **description** | **unit** |
| --- | --- | --- |
| *A_canopy_* | Integrated net daily carbon fixation rate of total canopy | (kg CH_2_O ha^-1^ day^-1^) |
| An | Net assimilation rate | µmol m^-2^ s^-1^ |
| *A_sun_*, *A_shade_* | Net assimilation rate of sunlit/shaded part of the canopy | (µmol m^-2^ s^-1^) |
| *C_1_…C_7_* | Empirical fit constants to derive saturated vapour pressure from temperature. | (Pa…Pa ^o^C^-6^) |
| *C_i_*, *C_a_* | Intercellular or ambient CO_2_ partial pressure | (Pa) |
| *Dj* (212) | Day of year | (-) |
| *e_s_* | saturated vapour pressure | (Pa) |
| *f_NJ_* | Fraction of total leaf N redistributed to Jmax | (-) |
| *f_NV_* | Fraction of total leaf N redistributed to Vcmax | (-) |
| *F_sun_, F_shade_, F_tot_* | Sunlit, shaded and total canopy leaf area index (m^2^ m^-2^) | m^2^ m^-2^ |
| *g_0_* (0.0811) | Ball-berry intercept | µmol Pa^-1^ m^-2^ s^-1^ |
| *g_1_* (7.5) | Ball-berry slope parameter | - |
| *g_s_* | Stomatal conductance to CO_2_ | µmol Pa^-1^ m^-2^ s^-1^ |
| *H_r_* (0.7) | Relative humidity at 25 ^o^C | - |
| *I_direct_*, *I_diffuse,_I_scat_* | Photon flux density of direct/diffuse/scattered irradiance | (µmol m^-2^ s^-1^) |
| *I_S_* (2600) | solar constant, i.e. the photon flux density in a plane perpendicular to the solar beam above the atmosphere | (µmol m^-2^ s^-1^) |
| *I_sun_*, *I_shade_* | Photon flux density incident on sunlit/shaded part of the canopy | (µmol m^-2^ s^-1^) |
| *J* | Rate of linear electron transport | (µmol m^-2^ s^-1^) |
| *J_max_* (205) | Maximal rate of linear electron transport | µmol m^-2^ s^-1^ |
| *k* | Foliar extinction coefficient | (-) |
| *K_c_* | Rubisco Michaelis constant for CO_2_ | (Pa) |
| *K_o_* | Rubisco Michaelis constant for O_2_ | (Pa) |
| *N_tot_* (2) | Total leaf nitrogen content | (g m^-2^) |
| *O* (21000) | atmospheric oxygen partial pressure, assumed to equal chloroplastic oxygen partial pressure | Pa |
| *P*, *P_o_* (1×10^5^) | Atmospheric pressure, Standard atmospheric pressure at sea level (Pa) |  |
| PFD | Incident quanta | (µmol m^-2^ s^-1^) |
| *Q_2_* | Incident quanta utilized in electron transport through PSII | (µmol m^-2^ s^-1^) |
| *Rd* (2) | Mitochondrial respiration rate not associated with photo-respiration | (µmol m^-2^ s^-1^) |
| *t, t_sn_* | time of day, time of solar noon | (h) |
| *V_cmax_* (130) | Maximal rate of RuBP carboxylation | µmol m^-2^ s^-1^ |
| *W_c_* | Rubisco limited rate of carboxylation | (µmol m^-2^ s^-1^) |
| *W_J_* | RuBP-limited rate of carboxylation | (µmol m^-2^ s^-1^) |
| x (1) | ratio of horizontal to vertical projected canopy leaf area | (-) |
| α (0.85) | atmospheric transmittance | (-) |
| α_l_ (0.85) | Leaf absorptance | (-) |
| β (0.5) | fraction of absorbed light that reaches photosystem II | (-) |
| *Γ** | CO_2_ compensation point in the absence of respiration | (Pa) |
| δ | Solar declination | (^o^) |
| θ | Solar zenith angle | (^o^) |
| θPSII (0.5) | Convexity of the non-rectangular curve describing the dependence of J on PFD | (-) |
| λ | Angle between leaf surface and the direct beam solar radiation | (^o^) |
| ΦPSIImax (0.83) | Maximal quantum yield of photosystem II | (-) |
| Ω (42.5) | Latitude | (^o^) |

**Reference list supplemental materials**

Ball JT, Woodrow IE, Berry JA 1987 A model predicting stomatal conductance and its contribution to the control of photosynthesis under different environmental conditions. In: Biggins, J (Ed.) Progress in Photosynthesis Research vol. 4. Proceedings of the 7^th^ international congress on Photosynthesis. Martins Nijhoff, Dordrecht, The Netherlands, pp221-224.

Collins M, Knutti R, Arblaster J, Dufresne JL, Fichelet T, Friedlingstein P, Gao, X, Gutowski WJ, Johns T, Krinner G, shongwe M, Tebaldi C, Weaver AJ, Wehner M. 2013 Long-term Climate Change: Projections, Commitments and Irreversibility. In: Climate Change 2013: The Physical Science Basis. Contribution of Working Group I to the Fifth Assessment Report of the Intergovernmental Panel on Climate Change. (Eds. Stocker TF, Qin D, Plattner GK, Tignor M, Allen SK, Boschung J, Nauels A, Xia Y, Bex V, and Midgley PM). Cambridge University Press, Cambridge, UK and NY USA.

Flatau PJ, Walko RL, Cotton WR 1992 Polynomial fits to Saturation Vapor Pressure. Journal of applied meteorology 31: 1507-1513.

Harley PC, Thomas RB, Reynolds JF, Strain BR 1992 Modelling photosynthesis of cotton grown in elevated CO_2_. Plant Cell and Environment 15: 271-282.

Jones PD, New M, Parker DE, Martin S and Rigor IG. 1999. Surface air temperature and its variations over the last 150 years. Reviews of Geophysics 37, 173-199.

Long SP, Bernacchi CJ 2003 Gas exchange measurements, what can they tell us about the underlying limitation to photosynthesis? Procedures and sources of error. Journal of Experimental Botany 54: 2393-2401.

Ort DR, Zhu XG, Melis A. 2010 Optimizing Antenna Size to Maximize Photosynthetic Efficiency. Plant Physiology 155: 79-85.

Sharkey TD, Bernacchi CJ, Farquhar GD, Sinqsaas EL 2007 Fitting photosynthetic carbon dioxide response curves for C(3) leaves. Plant Cell and Environment 30: 1035-40
